# CoVizu: Rapid analysis and visualization of the global diversity of SARS-CoV-2 genomes

**DOI:** 10.1093/ve/veab092

**Published:** 2021-11-08

**Authors:** Roux-Cil Ferreira, Emmanuel Wong, Gopi Gugan, Kaitlyn Wade, Molly Liu, Laura Muñoz Baena, Connor Chato, Bonnie Lu, Abayomi S Olabode, Art F Y Poon

**Affiliations:** Department of Pathology and Laboratory Medicine, Western University, London, ON, Canada; Department of Pathology and Laboratory Medicine, Western University, London, ON, Canada; Department of Pathology and Laboratory Medicine, Western University, London, ON, Canada; Department of Pathology and Laboratory Medicine, Western University, London, ON, Canada; Department of Pathology and Laboratory Medicine, Western University, London, ON, Canada; Department of Microbiology and Immunology, Western University, London, ON, Canada; Department of Pathology and Laboratory Medicine, Western University, London, ON, Canada; Department of Pathology and Laboratory Medicine, Western University, London, ON, Canada; Department of Pathology and Laboratory Medicine, Western University, London, ON, Canada; Department of Pathology and Laboratory Medicine, Western University, London, ON, Canada; Department of Microbiology and Immunology, Western University, London, ON, Canada

**Keywords:** SARS-CoV-2, genomic epidemiology, data visualization

## Abstract

Phylogenetics has played a pivotal role in the genomic epidemiology of severe acute respiratory syndrome coronavirus 2, such as tracking the emergence and global spread of variants and scientific communication. However, the rapid accumulation of genomic data from around the world—with over two million genomes currently available in the Global Initiative on Sharing All Influenza Data database—is testing the limits of standard phylogenetic methods. Here, we describe a new approach to rapidly analyze and visualize large numbers of SARS-CoV-2 genomes. Using Python, genomes are filtered for problematic sites, incomplete coverage, and excessive divergence from a strict molecular clock. All differences from the reference genome, including indels, are extracted using minimap2 and compactly stored as a set of features for each genome. For each Pango lineage (https://cov-lineages.org), we collapse genomes with identical features into ‘variants’, generate 100 bootstrap samples of the feature set union to generate weights, and compute the symmetric differences between the weighted feature sets for every pair of variants. The resulting distance matrices are used to generate neighbor-joining trees in RapidNJ that are converted into a majority-rule consensus tree for each lineage. Branches with support values below 50 per cent or mean lengths below 0.5 differences are collapsed, and tip labels on affected branches are mapped to internal nodes as directly sampled ancestral variants. Currently, we process about 2 million genomes in approximately 9 h on 52 cores. The resulting trees are visualized using the JavaScript framework D3.js as ‘beadplots’, in which variants are represented by horizontal line segments, annotated with beads representing samples by collection date. Variants are linked by vertical edges to represent branches in the consensus tree. These visualizations are published at https://filogeneti.ca/CoVizu. All source code was released under an MIT license at https://github.com/PoonLab/covizu.

## 1. Introduction

**Figure 1.**
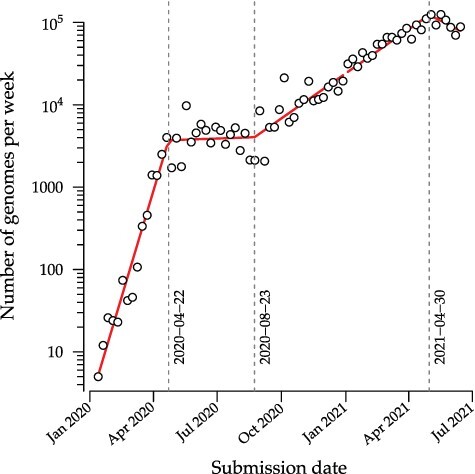
Weekly numbers of genomes submitted to the GISAID database (accessed on 26 June 2021). Red line segments represent the fit of a piecewise linear regression with three change points (indicated by vertical dashed lines) using the R package segmented (Muggeo 2008). An increasing linear trend relative to a log-transformed *y*-axis indicates an exponentially growing rate of genome submission.

Severe acute respiratory syndrome coronavirus 2 (SARS-CoV-2) was first sampled in December 2019, in association with an outbreak of unexplained pneumonia in the province of Hubei, China (Wu et al. 2020b). The first genome sequence of the novel coronavirus isolated from this outbreak was released into the public domain on 10 January 2020 (Wu et al. 2020a). Early phylogenetic analyses of this and subsequent genome samples provided initial evidence of human-to-human transmission (Rambaut and Andersen 2020) and estimates of the basic reproduction number (Riou and Althaus 2020). By the end of March 2020, over 200 countries had reported at least one confirmed case of SARS-CoV-2 from importation or local transmission, and a global pandemic was formally declared by the World Health Organization. One of the most remarkable developments from global efforts to control the pandemic has been the rapid accumulation and generally timely release of SARS-CoV-2 genome sequence data into the public domain. As of 21 June 2021, over 2 million SARS-CoV-2 genomes have been deposited in the Global Initiative on Sharing All Influenza Data (GISAID) database (Elbe and Buckland-Merrett 2017), and this number has grown at a sustained and exponentially increasing rate (Fig. 1). The phylogenetic analysis of these data has played an important role in tracking the genomic epidemiology of SARS-CoV-2. For example, Nextstrain (https://nextstrain.org, last accessed 22 October 2021; (Hadfield et al. 2018)) publishes time-scaled phylogenetic trees (Sagulenko et al. 2018) as interactive visual summaries of the diversity of SARS-CoV-2 genomes at global and local scales. The resulting web documents are updated in real time with the availability of new data. Throughout the SARS-CoV-2 pandemic, Nextstrain has featured prominently in global variant tracking and scientific communication, and in some cases it has directly influenced public health decision-making (Bedford et al. 2020).

However, the data visualization maintained by Nextstrain is limited in practice to fewer than about 5,000 genome sequences. This constraint is not only due to the computational complexity of reconstructing large trees by maximum likelihood, but also the general difficulty of displaying large trees in a limited visual space (e.g., a web browser window) in a meaningful and interpretable way. Accurately reconstructing a large phylogeny is difficult because the number of possible trees grows faster than exponentially with the number of observed sequences; however, the amount of phylogenetic signal in the data has a fixed upper limit since we cannot sequence more than the full-length genome. Furthermore, the time scale of transmission for SARS-CoV-2 tends to outpace its molecular clock, such that many new infections are genetically identical to their source populations. Paradoxically, we can become increasingly uncertain about the relationships among specific lineages as we collect greater amounts of data (Morel et al. 2021)). This uncertainty is exacerbated by sequencing error (Turakhia et al. 2020) and a substantial prevalence of missing data, i.e., incomplete genome sequences.

Even if it is computationally feasible to accurately infer the evolutionary relationships among millions of sampled infections, visualizing these results in a meaningful way is a significant challenge. A standard maximum likelihood phylogeny, for example, does not differentiate between samples with identical sequences. These samples become collapsed into a single node, even if they were collected on different dates or at different locations. Given these metadata, however, genetically identical samples carry epidemiologically relevant information at an individual level. While Bayesian methods can incorporate prior information to resolve the relationships between identical samples (Boskova and Stadler 2020), these methods are computationally demanding and the outputs do not necessarily result in an efficient or effective use of visual space.

Here, we describe an ongoing open-source project to provide a public interface to visualize the global diversity of SARS-CoV-2 genomes in near real time. Development of CoVizu (derived from ‘coronavirus visualization’) began in April 2020. From December 2020 onward, CoVizu became provisioned by a customized data feed from the GISAID database (https://gisaid.org, last accessed 22 October 2021), which is presently the largest publicly accessible repository of SARS-CoV-2 genome sequence data in the world. The specific objectives of this project are: (1) to process and visualize as much of the GISAID database as possible (i.e., millions of genomes); (2) to reconstruct robust evolutionary and epidemiological relationships among these genomes; (3) to continually update outputs with new genomic data as frequently as possible; and (4) to present this information in a rich and intuitive visual interface.

CoVizu is composed of a Python-based ‘backend’—an analytical pipeline for rapidly inferring the evolutionary relationships among genome sequences—and a JavaScript-based ‘frontend’ to visualize these relationships. It relies heavily on the manually curated Pango nomenclature system that partitions the global diversity of SARS-CoV-2 into a hierarchy of ‘lineages’ (Rambaut et al. 2020). The web interface is presently hosted at https://filogeneti.ca/CoVizu (last accessed 22 October 2021), and as an integrated component of the GISAID web portal. All Python and JavaScript source code comprising the project is publicly available from our repository (https://github.com/PoonLab/CoVizu, last accessed 22 October 2021) under the Massachusetts Institute of Technology (MIT) license.

## 2. Data analysis

The CoVizu backend is implemented in the Python scripting language. Raw sequence data and metadata, including sample collection dates and Pangolin (Rambaut et al. 2020) lineage assignments, are provisioned by the GISAID database as a single Lempel–Ziv–Markov compressed JSON (JavaScript Object Notation) file.

### 2.1 Sequence alignment and cleaning

An uncompressed data stream from the GISAID provisioned file is processed in Python to exclude any record whose genome sequence: (1) lacks a Pango lineage assignment; (2) was sampled from a non-human host; (3) was shorter than 29,000 nt; (4) lacks a complete sample collection date (e.g., year and month with no day); or (5) was labeled with a collection date preceding 1 December 2019 or in the future. The filtered data stream is then redirected to the program minimap2 (version 2.17; (Li 2018)) for pairwise alignment against the SARS-CoV-2 reference genome (GenBank accession NC_045512; (Wu et al. 2020a)). We parse the resulting SAM (sequence alignment/map) formatted output stream in Python to extract any genetic differences (nucleotide substitutions, insertions, and deletions) from the reference as ‘features’, as well as any intervals of uncalled bases (missing data). These provide a compact representation of each genome sequence. Any genomes that failed to map to the reference or contained over 300 (~1  per cent) uncalled bases are excluded at this stage. Furthermore, the feature set of each genome is screened for problematic sites using the curated list maintained at https://github.com/W-L/ProblematicSites_SARS-CoV2 (last accessed 22 October 2021; (De Maio et al. 2020)). We also exclude genomes where the number of features is either above the 99.9 % or below the 0.1 percentile of a Poisson distribution with rate parameter $\lambda = r \Delta t$, where *r* = 0.0655 substitutions/genome/day and $\Delta t$ is the number of days since 1 December 2019 (Rambaut 2020). We use the SciPy root-finding method (Virtanen et al. 2020) to numerically solve for the transition points of the Poisson cumulative distribution function at different values of $\Delta t$. The genome records that pass these filters are partitioned into a dictionary keyed by Pango lineage assignment.

### 2.2 Time-scaled tree reconstruction

To reconstruct a time-scaled phylogeny that relates the Pango lineages in the database, we select a single representative genome for each lineage. We use the curated list of genomes associated with Pango lineage designations (https://github.com/cov-lineages/pango-designation/blob/master/lineages.csv, last accessed 22 October 2021) to screen for candidates from all genomes passing all the above filtering criteria by matching sample names and then select the candidate with the earliest sample collection date. We reconstitute a multiple sequence alignment of these representative genomes from the respective feature sets by excluding all insertions relative to the reference genome. Next, we reconstruct a maximum likelihood tree using the fast approximate heuristic method implemented in FastTree (version 2.1.11, compiled for double precision; (Price et al. 2010)). Any internal nodes with a parametric bootstrap support below 50 per cent are collapsed into polytomies. We rescale the resulting tree using TreeTime (version 0.8.0; (Sagulenko et al. 2018)) with a pre-specified clock rate ($8\times 10^{-4}$ substitutions/site/year) and retaining polytomies. This final tree is processed using the Biopython Phylo submodule (Talevich et al. 2012) and serialized into the Newick tree format with terminal nodes labeled by Pango lineage.

### 2.3 Clustering analysis

We use the neighbor-joining method (Saitou and Nei 1987) to reconstruct the evolutionary relationships among genomes within each Pango lineage. To reduce computing time, all lineages with 5,000 samples or fewer are processed in a single batch distributed over multiple cores in a clustered computing environment using the Python-MPI (message passing interface) bindings implemented in the mpi4py module (Dalcín et al. 2008). Lineages with more than 5,000 samples are processed singly, with bootstrap replicates distributed over multiple cores. Neighbor-joining is a clustering method that requires a pairwise distance matrix as input. Because we want to incorporate indel variation while minimizing computation time, we assume a uniform rate over all possible genetic differences and ignore multiple hits. To minimize the memory consumption at this step, we convert the features into integers by indexing the ordered set union of all features observed for a given lineage. All genomes with identical feature sets are compressed into a single ‘variant’. This step tends to compress the number of samples by about 38 per cent on average (interquantile range 22–50 per cent), with a small number of variants comprising a disproportionately large number of samples. Since the time complexity of neighbor joining is $O(n^3)$ and memory consumption is $O(n^2)$ (Simonsen et al. 2008), we do not have the computational resources to process all available samples of a lineage at high global prevalence—such as B.1.617.2, also known as variant of concern ‘Delta’—in a reasonable amount of time. Moreover, tens of thousands of variants or more would be difficult to visualize meaningfully in any framework. Consequently, we sort the variants of a lineage by their most recent sample collection dates and retain only the most recent 5,000 variants. (The reduction in sample size by this step is reported in the web interface for each lineage.) The resulting dictionary of variants and indexed features, keyed by lineage, is serialized into a JSON file for parallel computation.

For each lineage, we compute the symmetric difference ($A\bigtriangleup B$) between the feature sets for every pair of variants, where $A\bigtriangleup B$ contains all features that are in either *A* or *B* but not both. For example, the symmetric difference between the subsets $A=\{1,4,128\}$ and $B=\{4,37,89\}$ is $A\bigtriangleup B=\{1,37,89,128\}$. To generate a bootstrap replicate, we sample the feature set union at random with replacement and use the resulting frequencies to weight the symmetric differences. Thus, the distance between *A* and *B* is given by $\sum_{i \in A\bigtriangleup B} f_b(i)$, where $f_b(i)$ is the frequency of the *i*-th feature in bootstrap replicate *b*. The resulting pairwise distance matrix is written to a comma-delimited file as input for neighbor-joining tree reconstruction using RapidNJ (version 2.3.2; (Simonsen et al. 2011)). We repeat this process for 100 bootstrap replicates.

We use a custom Python function to generate a consensus tree from all splits that occurred in at least 50 per cent of the bootstrap trees and assign branch lengths by averaging over the subset of trees containing each split. Next, we collapse any branches with a mean length below 0.5 features (genetic differences). If a terminal branch is collapsed, then its variant label is re-assigned to the parent internal node. If an internal branch is collapsed, then any variant labels carried by that node are reassigned to its parent. Thus, an internal node may be associated with multiple variants that are too genetically similar to be reproducibly distinguished. We interpret a labeled internal node as an ancestral variant that has been directly observed as a genome sequence. The resulting tree is serialized into a JSON file comprising node and edge lists. A node list is an associative array comprising lists of sample labels keyed by variant. An edge list comprises pairs of parent and child nodes (variants), branch lengths and bootstrap support values. Although this clustering method is designed to be fast and approximate, the resulting consensus trees have topologies and branch lengths concordant with maximum likelihood trees reconstructed from the same genomic data (e.g., see Supplementary Fig. S1).

## 3. Data visualization

**Figure 2.**
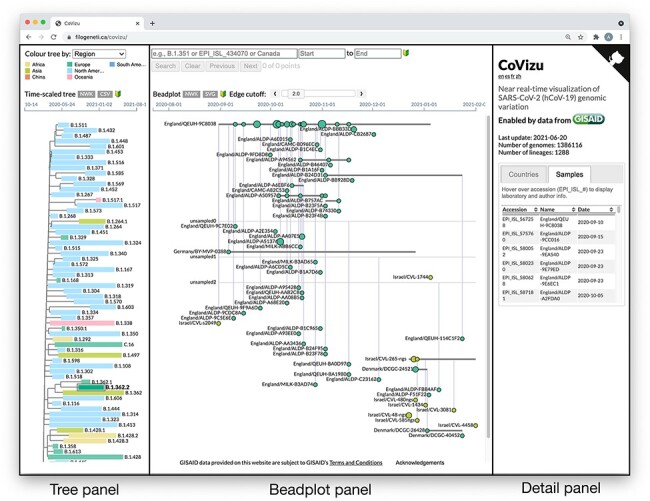
The CoVizu frontend presented as a single webpage at https://filogeneti.ca/CoVizu. Visual information is arranged into three panels (emphasized with rectangular boxes and labels) to present the data at decreasing levels of granularity from left to right. The leftmost panel displays a time-scaled tree relating Pango lineages, colored by geographic region in this instance. Selecting a lineage updates the middle panel to display a beadplot of its variants and samples. In this example, we have selected lineage B.1.362.2, which was sampled predominantly in Europe and comprised 99 samples grouped into 59 variants. The rightmost panel depicted here displays a scrollable table of sample accessions, names, and collection dates.

The CoVizu frontend is implemented in JavaScript using the D3.js (https://d3js.org/, last accessed 22 October 2021) and jQuery UI (https://jqueryui.com/, last accessed 22 October 2021) frameworks. Upon completion of the analysis pipeline, JSON data from the clustering analysis and the Newick file from the time-scaled tree reconstruction are automatically transferred from the computing cluster to the web server. To reduce page load time, the JSON data are transmitted to the client in a gzip-compressed format. These data are used to render SVG (support vector graphics) and HTML elements in three panels that represent different levels of data aggregation from left to right. The leftmost panel depicts the time-scaled tree relating Pango lineages and corresponds to the highest level of data aggregation. The middle panel depicts a ‘beadplot’ that we use to visualize the genetic variation within a selected Pango lineage. The width of the beadplot scales dynamically with the horizontal dimension of the browser window. Finally, the rightmost panel displays an interactive dynamic table that displays the individual samples for the selected lineage, variant, or bead. All these visual outputs are presented as a single composite webpage (Fig. 2). This webpage and its text components, e.g., pop-up dialogs, have been translated into French, Spanish, and Chinese.

### 3.1 Time-scaled tree

The time-scaled tree relating Pango lineages is rendered as an SVG using a rectangular layout algorithm, with the earliest time point on the left-hand side. Each tip representing a lineage is associated with a rectangular element spanning the range of sample collection dates. This visualization scheme was recently adopted by https://covidcg.org (last accessed 22 October 2021; (Chen et al. 2021)) for their lineage report interface. On CoVizu, the user can select whether the rectangles are colored according to the number of samples, most recent collection date, average deviation from the molecular clock, or the predominant geographical region of sampling (Africa, Asia, China, Europe, North America, Oceania, and South America). China is classified as a region because the samples from this country in the GISAID database are labeled by district (e.g., Guangzhou) instead of by country. We used a qualitative color-accessible palette developed by Paul Tol (https://personal.sron.nl/~pault/, last accessed 22 October 2021) for regions, and built-in D3.js palettes for other color schemes. The time-scaled tree can be downloaded by the user in the Newick tree format, and lineage-level statistics can be downloaded as a comma-separated values (CSV) formatted file.

Mouseover events on rectangular elements trigger a ‘tool tip’ dialog that provides lineage-level summary statistics, such as the number of samples and mean deviation from the clock model. In addition, this dialog displays a list of all mutations that were observed in at least 50 per cent of samples. Following the colon-delimited notation used in https://cov-lineages.org (last accessed 22 October 2021), amino acid substitutions are prefixed with ‘aa’ and labeled by the protein abbreviation and position in the reference protein sequence. For example, ‘aa:S:D614G’ represents a substitution of aspartic acid by glycine at Position 614 of the spike protein. Insertions and deletions are prefixed respectively with ‘ins’ and ‘del’, and labeled by the reference nucleotide coordinate and indel length in nucleotides. For example, ‘del:11288:9’ represents a deletion of nine nucleotides at genome coordinates 11288–11296 (inclusive).

### 3.2 Beadplots

**Figure 3.**
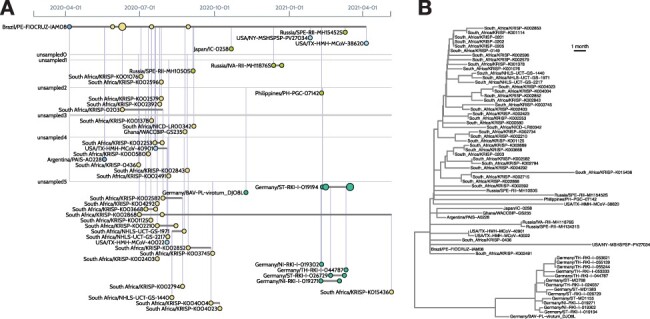
Visualizing the sample composition of a lineage. (A) The left image was generated by the CoVizu website for lineage B.1.1.117, using data retrieved from GISAID on 20 June 2021. Each horizontal line represents genomes that are indistinguishable in sequence (comprising a ‘variant’), labeled by the name of the earliest sample. For example, Brazil/PE-FIOCRUZ-IAM08 (upper left) was sampled on 6 April 2020, and identical genomes were subsequently observed in six samples in South Africa. This pattern is consistent with the importation of this variant from Brazil to South Africa. A more recent variant (Germany/ST-RKI-I-019194, seven samples) is ancestral to several other variants sampled in Germany (lower right). It is derived from an unsampled ancestral variant (‘unsampled2’) at a distance of 3.8 mutations, averaged over 100 bootstrap replicates, which in turn is separated from Brazil/PE-FIOCRUZ-IAM08 by a mean of 10.5 mutations. These lengths imply that this lineage is relatively undersampled. (B) For comparison, the right image depicts a time-scaled tree generated from the same data using FastTree2 and TreeTime. In this visualization, it is more difficult to identify samples that are genetically indistinguishable. Thus, beadplots endeavor to visually emphasize features that are relevant for public health applications.

Selecting a lineage in the time-scaled tree triggers the browser to render a beadplot (Fig. 3) in the middle panel as an SVG. A beadplot is a custom visual device that summarizes the distribution and genetic variation of samples within a lineage. The horizontal axis of the beadplot is scaled to the range of sample collection dates for the lineage. Samples with indistinguishable genome sequences are grouped into variants. In other words, each variant corresponds to a node in the consensus tree. (In the context of SARS-CoV-2, the term *variant* is often used interchangeably with *clade* or *lineage* (Mascola et al. 2021). However, *variant* can also refer to any unique combination of differences from a reference sequence.) Each variant is represented by a horizontal line segment in the beadplot.

Each horizontal line segment spans the range of sample collection dates. Circles (beads) along a line segment represent samples. The area of the bead is scaled in proportion to the number of samples collected on the same date. In addition, each circle is colored with respect to the most common geographic region of the samples. Together these elements provide an intuitive visual summary of the frequencies of a specific variant over time. Furthermore, sampling the same or closely related variants in different regions provides evidence of importation events (Fig. 3). Unsampled variants, which correspond to unlabeled internal nodes in the consensus tree, are represented by horizontal line segments that are not annotated with beads and span the entire width of the beadplot. The existence of these latent variants is inferred by the common ancestry of variants that are directly observed. Variants are connected by vertical line segments that correspond to branches in the consensus tree. Because the number of branches can become excessive for large beadplots, we provide a slider widget for users to filter branches by mean length. Horizontal lines can extend beyond the first and last samples of the corresponding variant if descendant variants are sampled at earlier or later dates, respectively.

All elements of a beadplot SVG are visually responsive to mouseover events, which also triggers a tooltip dialog summarizing variant- or bead-level summary statistics, including the number of samples, branch lengths, and the parent and child variants. The displayed beadplot can be exported as either a consensus tree (Newick format) or a SVG file, which can be converted into any rasterized or vector-based image format.

### 3.3 Sample details

**Figure 4.**
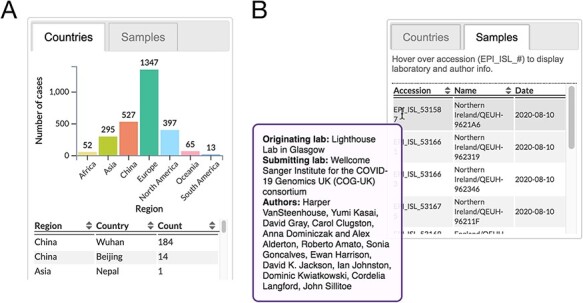
Excerpts from sample details panel. (A) An example of geographical metadata displayed for a lineage. (B) Table of sample information. Contributing laboratory and author information is retrieved from GISAID and displayed in a tooltip upon a mouseover event bound to accession numbers.

The rightmost panel of the web page presents some database-level statistics—namely, the date of the current update, total number of genomes that passed the quality filters, and the number of Pango lineages—and a tabbed content area where the user can switch between two interfaces that summarize the sample metadata. The ‘countries’ interface displays a bar plot that summarizes the distribution of samples among geographic regions, and a sortable table that breaks down these frequencies by country (Fig. 4A) for the selected element. The ‘samples’ interface, on the other hand, displays a sortable table that lists the accession number, name (label), and collection date for every sample associated with the selected element. In addition, mouseover events are bound to the accession numbers in the table to trigger a query of the GISAID API for retrieving laboratory and author information for the sample, which is displayed as a pop-up dialog (Fig. 4B).

### 3.4 Search interface

Since the frontend was designed to enable users to browse the relationships among millions of SARS-CoV-2 samples, we also needed to implement a search interface to enable users to quickly focus on samples matching specific parameters. The search interface comprises a text box for submitting a substring query, which can be matched against Pango lineage names, GISAID accession numbers, countries, and sample names, and date selection widgets for specifying a range of sample collection dates. If the substring query matches a regular expression that identifies it as a partial Pango lineage name or accession number, the browser populates a drop-down with suggested ‘autocompletions’ of the substring.

The submitted query is compared to metadata extracted from all samples, and the unique identifiers of bead and lineage elements that contain hits are stored. Next, the browser modifies the class attribute of all of matching elements, which causes the window to update how these elements are drawn, i.e., with CSS highlighting. Caching the search results in this way streamlines the process of navigating between lineages and rendering the corresponding beadplots. The total number of hits is displayed below the search interface. Finally, the user can traverse search results using either the ‘next’ and ‘previous’ buttons or arrow keys.

## 4. Concluding remarks

Over the course of the SARS-CoV-2 pandemic, it has quickly become clear that the standard phylogenetic toolkit was not up to the task of processing the overwhelming number of publicly accessible viral genomes collected around the world (Morel et al. 2021; Turakhia et al. 2020). This critical situation has catalyzed the development of new analytical methods (Boskova and Stadler 2020; Worobey et al. 2020). It has also led to the resurrection of classic methods in phylogenetics, including maximum parsimony (Turakhia et al. 2021) and, in our case, neighbor joining with an uncorrected distance. CoVizu is under continual development and many of the methods described here are subject to further enhancements and refactoring for improved performance. We welcome suggestions through our issue tracker for additional features, with the hope that this rapid analysis and visualization system can provide a unique, useful resource for public health monitoring and basic research.

## Supplementary data

Supplementary data is available at *Virus Evolution* online.

## Data Availability

All sequence data used to generate the figures are available at https://gisaid.org, using sample accession numbers provided in Supplementary Tables 1 and 2.

## Supplementary Material

veab092_Supp

## References

[R1] Bedford T. et al. (2020) ‘Cryptic Transmission of SARS-CoV-2 in Washington State’, *Science*, 370: 571–75. doi: 10.1126/science.abc0523 32913002 PMC7810035

[R2] Boskova V. and Stadler T. (2020) ‘PIQMEE: Bayesian Phylodynamic Method for Analysis of Large Data Sets with Duplicate Sequences’, *Molecular Biology and Evolution*, 37: 3061–75. doi: 10.1093/molbev/msaa136 32492139 PMC7530608

[R3] Chen A. T. et al. (2021) ‘COVID-19 CG Enables SARS-CoV-2 Mutation and Lineage Tracking by Locations and Dates of Interest’, *eLife*, 10: e63409. doi: 10.7554/eLife.63409 PMC7901870 33620031

[R4] Dalcín L. et al. (2008) ‘MPI for Python: Performance Improvements and MPI-2 Extensions’, *Journal of Parallel and Distributed Computing*, 68: 655–62. doi: 10.1016/j.jpdc.2007.09.005

[R5] De Maio N. et al. (2020) *Issues with SARS-CoV-2 Sequencing Data* <https://virological.org/t/issues-with-sars-cov-2-sequencing-data/473> accessed 22 Oct 2021.

[R6] Elbe S. and Buckland-Merrett G. (2017) ‘Data, Disease and Diplomacy: GISAID’s Innovative Contribution to Global Health’, *Global Challenges*, 1: 33–46. doi: 10.1002/gch2.1018 31565258 PMC6607375

[R7] Hadfield J. et al. (2018) ‘Nextstrain: Real-Time Tracking of Pathogen Evolution’, *Bioinformatics*, 34: 4121–3. doi: 10.1093/bioinformatics/bty407 29790939 PMC6247931

[R8] Li H. (2018) ‘Minimap2: Pairwise Alignment for Nucleotide Sequences’, *Bioinformatics*, 34: 3094–3100. doi: 10.1093/bioinformatics/bty191 29750242 PMC6137996

[R9] Mascola J. R., Graham B. S. and Fauci A. S. (2021) ‘SARS-CoV-2 Viral Variants – Tackling a Moving Target’, *JAMA*, 325: 1261–2. doi: 10.1001/jama.2021.2088 33571363

[R10] Morel B. et al. (2021) ‘Phylogenetic Analysis of SARS-CoV-2 Data is Difficult’, *Molecular Biology and Evolution*, 38: 1777–91. doi: 10.1093/molbev/msaa314 33316067 PMC7798910

[R11] Muggeo V. M. (2008) ‘Segmented: an R Package to Fit Regression Models with Broken-line Relationships’, *R News*, 8: 20–5.

[R12] Price M. N. et al. (2010) ‘Fasttree 2–Approximately Maximum-Likelihood Trees for Large Alignments’, *PLoS One*, 5: e9490. doi: 10.1371/journal.pone.0009490 PMC2835736 20224823

[R13] Rambaut A. (2020). Phylodynamic analysis | 176 genomes | 6 Mar 2020. <http://virological.org/t/phylodynamic-analysis-176-genomes-6-mar-2020/356> accessed 24 Nov 2020.

[R14] Rambaut A. and Andersen K. (2020). Preliminary phylogenetic analysis of 11 nCoV2019 genomes, <https://virological.org/t/preliminary-phylogenetic-analysis-of-11-ncov2019-genomes-2020-01-19/329/1> accessed 19 Jun 2020.

[R15] Rambaut A. et al. (2020) ‘A Dynamic Nomenclature Proposal for SARS-CoV-2 Lineages to Assist Genomic Epidemiology’, *Nature Microbiology*, 5: 1403–07. doi: 10.1038/s41564-020-0770-5 PMC7610519 32669681

[R16] Riou J. and Althaus C. L. (2020) ‘Pattern of Early Human-to-Human Transmission of Wuhan 2019f Wuhan 2019 Novel Corona Novel Coronavirus (2019-nCoV), December 2019 to January 2020’, *Eurosurveillance*, 25: 2000058. doi: 10.2807/1560-7917.ES.2020.25.4.2000058 PMC7001239 32019669

[R17] Sagulenko P. et al. (2018) ‘Treetime: Maximum-Likelihood Phylodynamic Analysis’, *Virus Evolution*, 4: vex042. doi: 10.1093/ve/vex042 PMC5758920 29340210

[R18] Saitou N. and Nei M. (1987) ‘The Neighbor-Joining Method: a New Method for Reconstructing Phylogenetic Trees’, *Molecular Biology and Evolution*, 4: 406–25. 3447015 10.1093/oxfordjournals.molbev.a040454

[R19] Simonsen M. et al. (2008) ‘Rapid Neighbour-Joining’ in, International Workshop on Algorithms in Bioinformatics, pp 113–22, Karlsruhe, Germany: Springer.

[R20] Simonsen M. et al. (2011) ‘Inference of Large Phylogenies Using Neighbour-Joining’ in, International Joint Conference on Biomedical Engineering Systems and Technologies, pp 334–44, Valencia, Spain: Springer.

[R21] Talevich E. et al. (2012) ‘Bio.Phylo: a Unified Toolkit for Processing, Analyzing and Visualizing Phylogenetic Trees in Biopython’, *BMC Bioinformatics*, 13: 1–9. doi: 10.1186/1471-2105-13-209 22909249 PMC3468381

[R22] Turakhia Y. et al. (2020) ‘Stability of Sars-Cov-2 Phylogenies’, *PLoS Genetics*, 16: e1009175. doi: 10.1371/journal.pgen.1009175 PMC7721162 33206635

[R23] Turakhias Y. et al. (2021) ‘Ultrafast Sample Placement on Existing tRees (UShER) Enables Real-Time Phylogenetics for the SARS-CoV-2 Pandemic’, *Nature Genetics*, 53: 809–16. doi: 10.1038/s41588-021-00862-7 33972780 PMC9248294

[R24] Virtanen P. et al. (2020) ‘SciPy 1.0: Fundamental Algorithms for Scientific Computing in Python’, *Nature Methods*, 17: 261–72. doi: 10.1038/s41592-019-0686-2 32015543 PMC7056644

[R25] Worobey M. et al. (2020) ‘The Emergence of SARS-CoV-2 in Europe and North America’, *Science*, New York, NY, 370: 564–70. doi: 10.1126/science.abc8169 32912998 PMC7810038

[R26] Wu F. et al. (2020a) ‘A New Coronavirus Associated with Human Respiratory Disease in China’, *Nature*, 579: 265–9. doi: 10.1038/s41586-020-2008-3 32015508 PMC7094943

[R27] Wu P. et al. (2020b) ‘Real-time Tentative Assessment of the Epidemiological Characteristics of Novel Coronavirus Infections in Wuhan, China, as at 22 January 2020’, *Eurosurveillance*, 25: 2000044. doi: 10.2807/1560-7917.ES.2020.25.3.2000044 PMC6988272 31992388

