# Supplementary figures and images for: CoVizu: Rapid analysis and visualization of the global diversity of SARS-CoV-2 genomes
Source: Virus Evol. 2021 Nov 8;7(2):veab092. doi: 10.1093/ve/veab092 (PMC10131274; doi:10.1093/ve/veab092)

# Consensus NJ

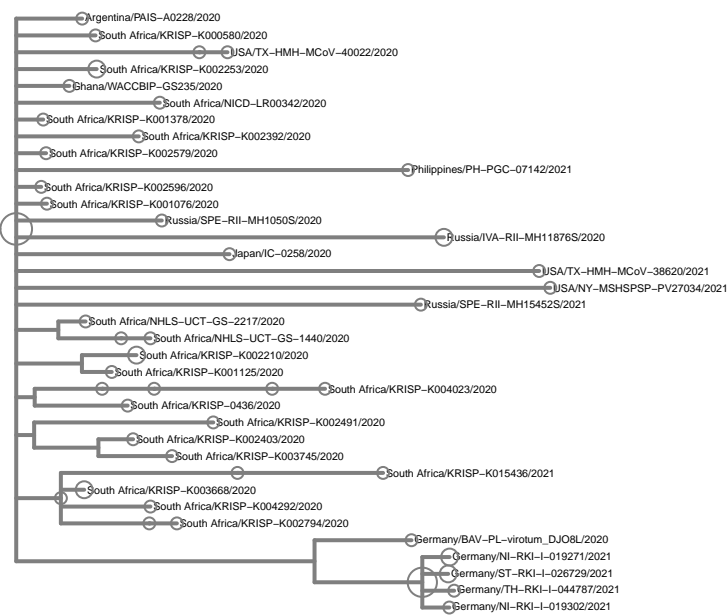

# Maximum likelihood

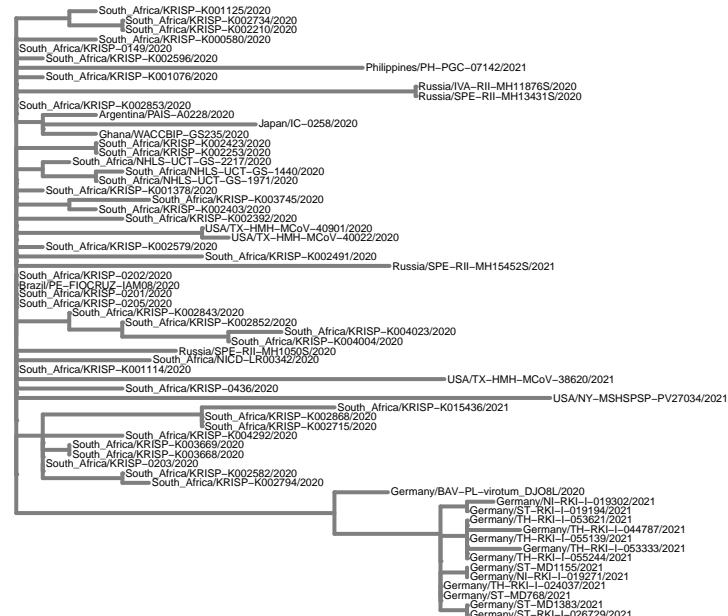

Terminal branch lengths, consensus NJ

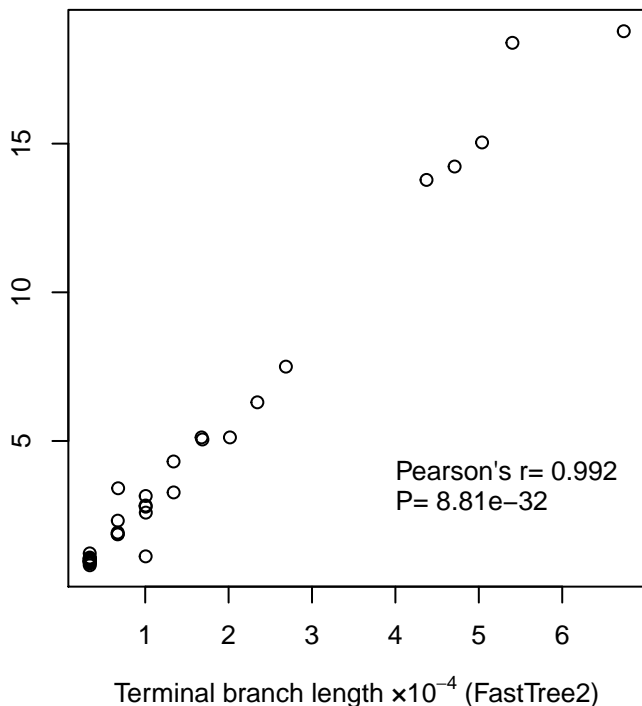

Supplement: veab092_Supp [file veab092_supp.zip › suppl_data/supfig.pdf]
